# Standardization and application of real-time polymerase chain reaction for rapid detection of bluetongue virus

**DOI:** 10.14202/vetworld.2018.452-458

**Published:** 2018-04-10

**Authors:** I. Karthika Lakshmi, Kalyani Putty, Satya Samparna Raut, Sunil R. Patil, P. P. Rao, B. Bhagyalakshmi, Y. Krishna Jyothi, B. Susmitha, Y. Vishnuvardhan Reddy, Sowmya Kasulanati, J. Shiva Jyothi, Y. N. Reddy

**Affiliations:** 1 Department of Bacteriology and Mycology, Veterinary Biological and Research Institute, Labbipeta, Vijayawada - 520 010, Andhra Pradesh, India; 2 Department of Veterinary Microbiology and Biotechnology, College of Veterinary Science, PVNRT Veterinary University, Hyderabad - 500 030, Telangana, India; 3 Ella Foundation, Genome Valley, Turkapally, Shameerpet Mandal, Hyderabad - 500 078, Telangana, India; 4 Department of Virology, Veterinary Biological and Research Institute, Labbipeta, Vijayawada - 520 010, Andhra Pradesh, India

**Keywords:** bluetongue virus, limit of detection, real-time polymerase chain reaction

## Abstract

**Aim:**

The present study was designed to standardize real-time polymerase chain reaction (PCR) for detecting the bluetongue virus from blood samples of sheep collected during outbreaks of bluetongue disease in the year 2014 in Andhra Pradesh and Telangana states of India.

**Materials and Methods:**

A 10-fold serial dilution of Plasmid PUC59 with bluetongue virus (BTV) NS3 insert was used to plot the standard curve. BHK-21 and KC cells were used for *in vitro* propagation of virus BTV-9 at a TCID50/ml of 10^5^ ml and RNA was isolated by the Trizol method. Both reverse transcription-PCR and real-time PCR using TaqMan probe were carried out with RNA extracted from virus-spiked culture medium and blood to compare the sensitivity by means of finding out the limit of detection (LoD). The results were verified by inoculating the detected and undetected dilutions onto cell cultures with further cytological (cytopathic effect) and molecular confirmation (by BTV-NS1 group-specific PCR). The standardized technique was then applied to field samples (blood) for detecting BTV.

**Results:**

The slope of the standard curve obtained was −3.23, and the efficiency was 103%. The LoD with RT-PCR was 8.269E×10^3^ number of copies of plasmid, whereas it was 13 with real-time PCR for plasmid dilutions. Similarly, LoD was determined for virus-spiked culture medium, and blood with both the types of PCR and the values were 10^3^ TCID 50/ml and 10^4^ TCID 50/ml with RT-PCR and 10° TCID 50/ml and 10^2^ TCID 50/ml with real-time PCR, respectively. The standardized technique was applied to blood samples collected from BTV suspected animals; 10 among 20 samples were found positive with Cq values ranging from 27 to 39. The Cq value exhibiting samples were further processed in cell cultures and were confirmed to be BT positive. Likewise, Cq undetected samples on processing in cell cultures turned out to be BTV negative.

**Conclusion:**

Real-time PCR was found to be a very sensitive as well as reliable method to detect BTV present in different types of samples, including blood samples collected from BTV-infected sheep, compared to RT-PCR. The LoD of BTV is likely influenced by sample type, possibly by the interference by the other components present in the sample.

## Introduction

Bluetongue (BT) is a viral disease which affects domestic and wild ruminants and is caused by BT virus (BTV), a dsRNA virus of the genus *Orbivirus* of the family Reoviridae [1]. It is a non-contagious disease that is primarily transmitted by adult midges belonging to *Culicoides* spp. [2]. However, recently, certain strains/serotypes are found to be transmitted horizontally [3]. The replication cycle in the insect is completed between 6 and 8 days [2]. Infected midges, blood, and semen act as sources of the virus. Although BTV has many vertebrate hosts including goats, cattle, buffaloes, and deer, the clinical manifestation of the disease is seen mainly apparent in sheep with signs such as fever, edema of the lips, tongue, and head, conjunctivitis, coronitis, excessive salivation, and nasal discharge [4]. The manifestation of the disease depends on serotype, species, breed, and age of animal with morbidity that could reach up to 100% and mortality between 30% and 70% [5]. Through transplacental transfer, the virus is also capable of exerting teratogenic effects, or the infection may lead to abortion in the pregnant host [6,7]. Curtailing the initial introduction into regions which harbor susceptible host and vector species and vaccination of the susceptible animal species may aid in effective prevention and control of BT [8].

At present, 27 serotypes of BTV have been recognized [4]. New serotypes of BTV are still emerging. A very flexible reassortment involving any of the genomic segment contributes majorly to the observed phenotypic variations in the BTV strains [9]. Although the VP2 protein encoded by seg-2 being highly variable is the determinant of serotype, the other viral protein VP5 is now found to codetermine serotype along with VP2 [10]. Diagnosis of BT requires isolation of virus, standard serological methods, and certain other antigen and nucleic acid detection assays [11]. Efficient diagnostic systems are required not only for the sensitive detection of BTV in clinical samples but also for the declaration/confirmation of virus-free status of non-endemic regions of the world. The serological tests used for the diagnosis of BTV include agar gel immunodiffusion, cELISA, and indirect ELISA, among which cELISA is the most preferred and reliable [2]. BTV is detected and isolated routinely, by direct inoculation on to cultured mammalian or insect cells, through the intravenous route into 10-12 days’ embryonated chicken eggs, followed by one passage in insect cell culture and up to three passages in mammalian cell cultures [12-14]. However, both the cell culture-based methods and serological methods are labor intensive, cost ineffective, and time taking. With interesting and desirable properties such as speed, high specificity, sensitivity, cost-effectiveness, and reduced contamination risk, polymerase chain reaction (PCR) has evolved as a very convenient alternative to the conventional methods of BTV detection. By targeting any of the several conserved BTV genome segments, such as those encoding VP1, VP3, VP7, NS1, NS2, and NS3, reverse transcription-PCR and real-time RT PCR assays are carried out for detecting BTV [14,15-20]. The present study is carried out with the objective of finding out the sensitivity of real-time RT PCR with different sources of viral RNA including BT suspected field samples.

## Materials and Methods

### Ethical approval

Consent was obtained from animal owners for blood collection from bluetongue suspected animals. Collection of blood samples was approved by Institutional animal ethics committee of PVNR TVU.

### Virus

BTV was isolated employing culture techniques using KC cell line and BHK-21 clone 13 cells [21]. For standardizing the technique, a BHK-21 T75 cm^2^ flask with about 70-80% monolayer was infected with plaque purified BTV9 isolated during outbreak of 2000 with a titer (TCID50) of 10^5^/ml.

### Plasmid and virus-spiked cell culture media, and virus-spiked blood

Plasmid PUC59 with NS3 gene insert that has a molecular weight of 0.4×10^−9^ ng was used. The concentration and purity of sample were 460 µg/ml and 1.933, respectively, with copy no 5×10^9^. A 10-fold dilution and 10 such serial dilutions of the plasmid were used as a positive reference. BTV-9 at a concentration of 10^5^ TCID 50/ml was diluted 10-folds, and a total of 10 such dilutions were done. Virus-spiked sheep blood (1 ml) was centrifuged at 4000 rpm for 10 min at 4°C. Plasma was aspirated and cell pellet was washed with 1 ml of 1× PBS; supernatant was discarded carefully. Dilutions were done with some modifications where initial dilution was made with media and to the washed blood pellet (250 µl), this diluted virus was added and subsequent dilutions were made.

### Extraction of viral nucleic acid

Viral RNA was isolated from virus-spiked blood and cell culture dilutions using Trizol method [22]. RNA was also isolated from a total of 20 field samples collected during outbreaks of 2014. Briefly, 100-250 µl of blood sample was taken in 1.5 ml Eppendorf tube and centrifuged at 1000 g for 10 min at 4°C. Plasma was discarded and cell pellet was washed with 1 ml sterile PBS (pH 7.2) by centrifugation at 1000 g for 10 min at 4°C. Supernatant was discarded and washing was repeated twice. To the cell pellet, 750 µl of Trizol LS reagent was added and RNA was extracted as above.

### Standardization of RT-PCR and real-time PCR

#### RT-PCR

Reverse transcription for first strand cDNA synthesis was carried out with 20 µl reaction mixture containing MuMLV-reverse transcriptase enzyme (200 U/µl) (Invitrogen, Cat No. 28025-013), 10mM dNTPs, and NS3 primers. Primers for BTV NS3 with sequences F’TTGGAYAAAGCRATGTCAAA and R’ ACRTCATCACGAAACGCTTC (R=A+G, Y=C+T) (obtained in lyophilized form from SIGMA-ALDRICH) were used at a final concentration of 1 pmol/µl [23].

#### Reverse transcription

An RNA mix was prepared by mixing RNA isolated with dNTPs and primers. This RNA mix was heated at 65°C for 5 min and snap cooled on ice. To the RNA mix, RT mix prepared from 5× first strand buffer and MuMLV-reverse transcriptase was added. The RNA mix and RT mix were incubated in thermocycler under the following conditions: 25°C for 10 min; 42°C for 1 h followed by 70°C for 10 min.

#### PCR

Using the cDNA synthesized, PCR was carried out by preparing a master mix for a 10 µl reaction with the primers described above and using Taq polymerase. The reaction mixture and thermal cycling conditions were standardized at an initial denaturation of 94°C for 3 min, followed by 35 cycles of 94°C for 30 s, 55°C for 30 s, 72°C for 1 min, and final extension for 10 min at 72°C. The reaction products were analyzed on 2% agarose gel.

### Real-time PCR

#### Probe

Light cycler^®^ 480 Probe Master of 2× concentration (Cat. No. 04707494001) was used. It contains FastStart Taq DNA Polymerase, reaction buffer, dNTP mix, and 6.4 mM MgCl_2_. The probe used in the current standardized protocol was TaqMan probe. The oligo sequence 5´ [6FAM] ARGCTGCATTCGCATCGTACGC [TAM] 3´ has been labeled with reporter FAM (Carboxyfluorescein) at 5´ end and quencher Tamra at 3´ end. The oligo sequence is a part of segment 10 (NS3), the conserved region present in all 1-26 BTV serotypes. The probe was used at a final concentration of 0.2 µM [23]. The primers that were used for RT-PCR as mentioned above were used at a final concentration of 0.4 µM. The cDNA prepared from RNA of virus-spiked blood and culture medium as well as field samples were diluted five-fold and used as template, and amplification curves were obtained.

#### Thermocycler and cycling conditions

The reaction was carried out in light cycler^®^ 480 System from Roche Applied Biosystems. The cycling conditions are pre-incubation at 95°C for 2 min, initial denaturation at 95°C for 30 s, annealing at 56°C for 30 s, extension at 72°C for 30 s, and a final extension temperature of 72°C for 10 min.

#### Plasmid dilutions and standard curve

Ten-fold serial dilution of the plasmid starting with a concentration of 1.195E×10^9^ was carried out. These were used as standards for obtaining a standard curve.

## Results

### RT-PCR

**Figure-1:**
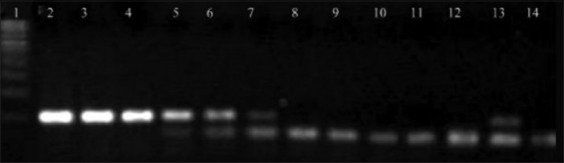
Standardization of RT-polymerase chain reaction in plasmid dilutions. Lane 1 - 100 bp ladder, lane 2 - 10^−1^ dilution; lane 3 - 10^−2^ dilution, lane 4 - 10^−3^ dilution, lane 5 - 10^−4^ dilution, lane 6 - 10^−5^ dilution, lane 7 - 10^−6^ dilution, lane 8 - 10^−7^ dilution, lane 9 - 10^−8^ dilution, lane 10 - 10^−9^ dilution, lane 11 - 10^−10^ dilution, lane 12 - 10^−11^dilution, lane 13 - positive control, lane 14 - negative control.

**Figure-2:**
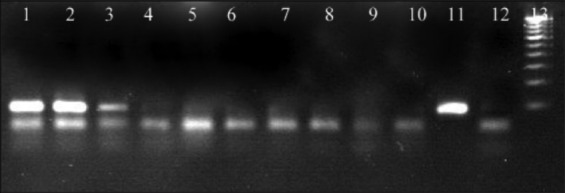
Standardization of RT-polymerase chain reaction in dilutions of virus-spiked cell culture media. Lane 1 - 10^5^ TCID50/ml, lane 2 - 10^4^ TCID50/ml; lane 3 - 10^3^ TCID50/ml, lane 4 - 10^2^ TCID50/ml, lane 5 - 10^1^ TCID50/ml, lane 6 - 10° TCID50/ml, lane 7 - 10^−1^ TCID50/ml, Lane 8 - 10^−2^ TCID50/ml, lane 9 - 10^−3^ TCID50/ml, lane 10 - 10^−4^ TCID50/ml, lane 11 - positive control, lane 12 - negative control, lane 13 - ladder.

**Figure-3:**
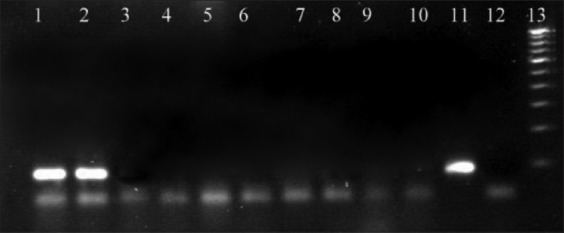
Standardization of RT-polymerase chain reaction in dilutions of virus-spiked blood. Lane 1 - 10^5^ TCID50/ml, lane 2 - 10^4^ TCID50/ml, lane 3 - 10^3^ TCID50/ml, lane 4 - 10^2^ TCID50/ml, lane 5 - 10^1^ TCID50/ml, lane 6 - 10^0^ TCID50/ml, lane 7 - 10^−1^ TCID50/ml, lane 8 - 10^−2^ TCID50/ml, lane 9 - 10^−3^ TCID50/ml, lane 10 - 10^−4^ TCID50/ml, lane 11 - positive control, lane 12 - negative control, lane 13 - ladder.

When RT-PCR was carried out with RNA extracted from plasmid dilutions, the limit of detection (LoD) was up to 6^th^ dilution, i.e., 8.269E×10^3^ number of copies of plasmid (Figure-1), whereas the LoD determined for virus-spiked culture medium and blood was 10^3^ TCID50/ml and 10^4^ TCID 50/ml, respectively (Figures-2 and 3).

### Real-time PCR

**Figure-4:**
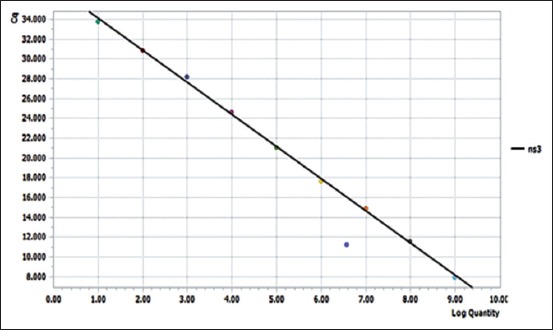
A standard curve with plasmid dilutions. A standard curve with a slope of −3.24, y-intercept 37.36, and efficiency obtained using the formula E=10^[–1/slope]^ was 103%. The plasmid dilutions Cq values are not deviating from the mean.

**Figure-5:**
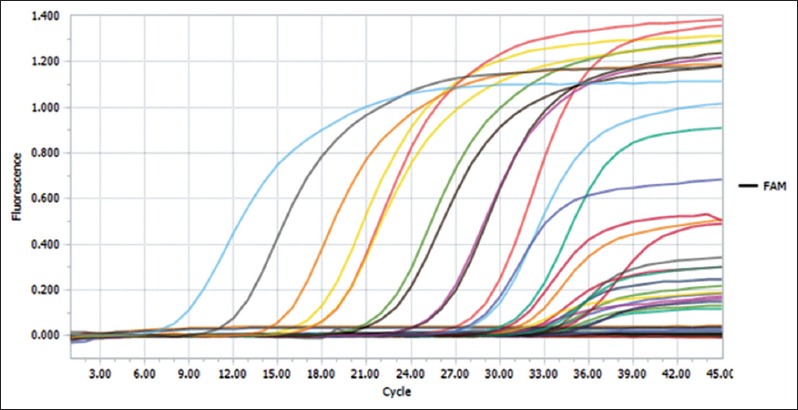
Amplification curve obtained by real-time polymerase chain reaction. Serial 10-fold dilutions of plasmid and virus-spiked blood and medium as a template. A specific set of primers and a FAM/TAMRA-labeled hydrolysis probes that recognize a 100 bp fragment of the NS3 gene were used. Although amplification curve is given separately for field samples, the determination of Cq values for the field samples tested was done based on the standard curve only.

A set of 10 serial dilutions of NS3 cloned plasmid were made and used as standards for development of a standard curve (Figure-4). Cq values obtained were increasing with increase in dilution of plasmid (Table-1) and ranged from 7.9 to 33.7. The least copy number that was able to be detected was 13. Cq values were plotted against the logarithm of the dilution factors, were found to not deviate much from the mean, and were falling on or near to mean line. Slope and efficiency of curve were −3.23 and 103%. Amplification curves were obtained for plasmid dilutions as well as virus-spiked culture medium and blood (Figure-5).LoD for virus-spiked culture medium and blood was found to be 10° TCID50/ml and 10^2^ TCID50/ml, respectively (Table-2).

**Table-1:** Cycle threshold values for plasmid dilutions.

Plasmid dilutions	C_q_	Concentration
P_1_	7.93	1.195E×10^9^
P_2_	11.56	9.073E×10^7^
P_3_	14.89	8.525E×10^6^
P_4_	17.63	1.1218E×10^6^
P_5_	21.01	1.105E×10^5^
P_6_	24.60	8.629E×10^3^
P_7_	28.16	6.886E×10^2^
P_8_	30.85	1.019E×10^2^
P_9_	33.72	1.328E×10^1^
P_10_	NA	NA

**Table-2:** Cycle threshold values for virus-spiked dilutions with medium and blood.

Dilution	TCID50 titer	Cq valuesvirus - spiked with medium and dilutions	Cq valuesvirus - spiked with blood and dilutions
1	10^5^	23	20.7
2	10^4^	25	27
3	10^3^	27	34.1
4	10^2^	28	36
5	10^1^	34	NA
6	10^0^	34.7	NA
7	10^−1^	NA	NA
8	10^−2^	NA	NA
9	10^−3^	NA	NA
10	10^−4^	NA	NA

### Application of real-time PCR for detection of BTV in field samples

**Figure-6:**
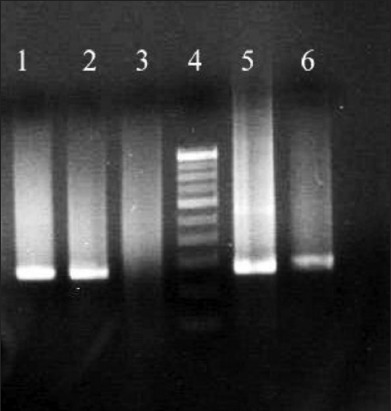
Molecular confirmation of Cq exhibiting samples as bluetongue virus positive following cell culture propagation. NS1 (274 bp) polymerase chain reaction for cell culture isolated from selected field samples are shown here. Similar results were seen with all the Cq value exhibiting samples used in the study. Lane 1 - 10MBNR, lane 2 - 5MMNR, lane 3 - negative control, lane 4 - 100 bp ladder, lane 5 - positive control, lane 6 - K4.

The standardized technique was then applied to detect BTV from blood samples collected from BT suspected animals. 10 among 20 samples were found positive with real-time PCR with Cq values ranging from 27 to 39 (Table-3). Cq values above 40 were considered as negative. None of these samples were tested positive by conventional RT-PCR. All the positive tested samples were further processed in cell cultures (KC and BHK 21), and RNA was isolated and has shown amplification with primers specific for NS1 (Figure-6).

**Table-3:** Cycle threshold values for field samples tested.

Sample	Cq
K4/15	27.38
10MBNR	34.82
5MMNR	30.47
4GDK/15	39.636
6GDK15	35.5
K2/15-	ND
K7/15	37.09
2PBR/15-	ND
7PBR/15	ND
12PBR	37
2PLR15	36.6
4PLR/15-	ND
2PKL/15	31.15
2MBNR/13	ND
4MBNR/13	ND
5MBNR/13	ND
6MBNR/13	ND
7MBNR/13	36.99
8MBNR/13	ND
537	ND
5MBNR/14	ND
PC	7.89
NC	ND

PC=Positive control, NC=Negative control, ND=Not determined

## Discussion

Employing immunological methods for detection of BTV has shortcomings such as the inability to detect in animals with low viremia and cross-reactivity with proteins from other Orbiviruses (excluding cELISA.) [24-26]. To circumvent these problems, PCR-based assays were explored for the reliable detection of BTV using different genomic segments [16-19]. The present study is aimed at developing a sensitive assay that is based on real-time PCR to help accelerate the process of detection as the routine methods such as inoculation of embryonated chicken eggs and/or cell cultures are time taking, as well as to enable detection of very low levels of BT viral load that is present in many field samples in BTV endemic areas or zones. The PCR assay is set up targeting the most conserved region of the BTV genome, and the segment 10 that encodes NS3 protein that is required for the release of newly made virions from infected cells [27]. For this, a serial 10-fold dilution of a plasmid PUC59 carrying NS3 of the viral genome as insert was taken and used for plotting the standard curve and amplification. The uniformity of these dilutions is evident from the gradual increase in the Cq value with increasing dilution. LoD was determined for both RT PCR and quantitative RT-PCR (qRT-PCR) methods for virus-spiked cell culture fluid, and blood as routine diagnosis is done majorly with blood samples. It is very much clear from the results of these assays that qRT-PCR is relatively more sensitive with LoD considerably lower than that of RT-PCR which is very much evident from the amplification curves obtained with viral RNA isolated from different types of samples, namely., blood and medium. Although standard curve with plasmid dilutions was limited to 10 copy numbers in the current study (with a Ct value of 34), and since Ct values as high as 39.636 were positive for BTV, setting up 2-fold dilutions for standard curve, especially at lower dilutions of plasmid, shall help in better interpretation of assay. However, it has to be also considered that with pure plasmid DNA molecules, when qRT-PCR was carried out, copies as low as 10 could be detected. However, the same number of copies in field blood samples may not be easily detected due to inhibitors that are likely to be present in the biological samples. Hence, field samples with higher Ct values were considered in the current study. The study also involved verification and confirmation of the assay results, for which the sample dilutions that marked no amplification and amplification were taken and inoculated onto cell cultures (KC and BHK-21 cell lines), and interestingly, no CPE and BT typical CPE, respectively, were observed in such cultures confirming the results obtained from the assay. Since the study was carried out with suspected field samples, the results and/or findings from the study can be directly adapted in laboratories that are involved in regular diagnosis of samples for BTV. Such early detection of virus present at very low level helps in implementing strategies such as vaccination to curtail further outbreaks to regions in proximity to the endemic areas.

## Conclusion

It can be concluded from the results of the study that real-time PCR stands out as a very sensitive and reliable assay for the detection of BTV present in different types of samples, with sensitivity varying with the type of sample.

## Author’s Contributions

KP, YNR, and PPR designed the study. KL, SSR, SRP, BB, KJY, SB, VRY, and SJ performed the experiments. KP, KL, and SK analyzed the data and wrote the manuscript.All authors read and approved the manuscript.
